# Single Crystalline Higher Manganese Silicide Nanowire Arrays with Outstanding Physical Properties through Double Tube Chemical Vapor Deposition

**DOI:** 10.3390/nano10091880

**Published:** 2020-09-19

**Authors:** Chin-Li Shen, Shu-Meng Yang, Kuo-Chang Lu

**Affiliations:** 1 Department of Materials Science and Engineering, National Cheng Kung University, Tainan 701, Taiwan; zzyzx1211@gmail.com (C.-L.S.); young263263@gmail.com (S.-M.Y.); 2 Center for Micro/Nano Science and Technology, National Cheng Kung University, Tainan 701, Taiwan

**Keywords:** higher manganese silicide, chemical vapor deposition, nanowires, transmission electron microscopy, ferromagnetism, field emission

## Abstract

In this work, we report a novel and efficient silicidation method to synthesize higher manganese silicide (HMS) nanowires with interesting characterization and physical properties. High density silicon nanowire arrays fabricated by chemical etching reacted with MnCl_2_ precursor through a unique double tube chemical vapor deposition (CVD) system, where we could enhance the vapor pressure of the precursor and provide stable Mn vapor with a sealing effect. It is crucial that the method enables the efficient formation of high quality higher manganese silicide nanowires without a change in morphology and aspect ratio during the process. X-ray diffraction (XRD), scanning electron microscopy (SEM) and transmission electron microscopy (TEM) were utilized to characterize the HMS nanowires. High-resolution TEM studies confirm that the HMS nanowires were single crystalline Mn_27_Si_47_ nanowires of Nowotny Chimney Ladder crystal structures. Magnetic property measurements show that the Mn_27_Si_47_ nanowire arrays were ferromagnetic at room temperature with a Curie temperature of over 300 K, highly depending on the relationship between the direction of the applied electric field and the axial direction of the standing nanowire arrays. Field emission measurements indicate that the 20 μm long nanowires possessed a field enhancement factor of 3307. The excellent physical properties of the HMS nanowires (NWs) make them attractive choices for applications in spintronic devices and field emitters.

## 1. Introduction

Transition metal silicide nanowires have been widely studied [1,2,3,4,5,6,7,8,9]. Fascinating materials from them with diverse features are recognized to be excellent candidates in various applications, including complementary metal-oxide semiconductor devices [10,11], electronics [12,13], photovoltaics [14,15], spintronics [16,17], field emission [18,19,20] and thermoelectrics [19,20,21,22,23]. Manganese silicides have been highly explored due to their interesting physical properties and potential applications. Additionally, semiconducting higher manganese silicides (HMS) are of a direct band gap of about 0.7 eV, suitable for thermoelectric [24,25,26,27] and optoelectronic [28] applications and having complicated phase characteristics and crystal structures. Higher manganese silicides represented by MnSi_2-x_, MnSi_1.75_ or MnSi_1.8_ are excellent thermoelectric materials due to their low thermal conductivity leading to a figure of merit of 0.7–0.8 [29,30]. HMS are of Nowotny Chimney Ladder (NCL) structures, described as chimneys of manganese atoms with silicon atoms, belonging to homologous series of specific HMS phases [21]. These crystal structures are composed of a tetragonal sublattice of Mn atoms and a sublattice of Si atoms, but the two sublattices have a slight mismatch in the c-direction of the crystal structure. Eight distinct phases with stoichiometry close to MnSi_1.75_ have been obtained in previous studies, including Mn_4_Si_7_, Mn_11_Si_19_, Mn_15_Si_26_, Mn_27_Si_47_, Mn_7_Si_12_, Mn_19_Si_33_, Mn_26_Si_45_ and Mn_39_Si_68_.

Single crystalline HMS nanowires and nanoribbons have been synthesized using chemical vapor deposition (CVD) with a single-source precursor of Mn(CO)_5_SiCl_3_ [15]; however, it is difficult to fabricate homemade precursors with a high yield. Recently, some interesting pathways synthesized by thermal evaporation and Si nanostructures have been explored [22,31,32]; nevertheless, the conversion of Si nanostructures into HMS nanostructures by precursor vapor has not been completely studied. Moreover, it is hard for the Si nanowires to react with precursor vapor of Mn source and to completely form HMS nanowires with the original nanoscale morphology preserved. In this study, as-prepared Si nanowires fabricated by chemical etching of Si substrate reacted with the precursor of MnCl_2_ through the double tube CVD method. We increased the precursor vapor with sufficient vapor pressure via sealing effect in the double tube system. The method facilitated efficient synthesis of high-quality HMS nanowires.

## 2. Experimental Section

### 2.1. Preparation of Si Nanowire Arrays

Si nanowire arrays were fabricated by silver-assisted electroless wet chemical etching as previously reported [33,34,35]. Clean substrates were etched in diluted HF (Honeywell FLUKA, Offenbach, Germany) to remove the native oxide layer, then put into a mixed solution of AgNO_3_ (Honeywell FLUKA, Offenbach, Germany) and HF at 40 °C for 50 min. The extra byproduct of Ag particle from the etching reaction was removed with HNO_3_ (Honeywell FLUKA, Offenbach, Germany) solution. Si nanowire arrays of approximately 20 μm in length and 50–150 nm in diameter were used for the following silicidation reaction.

### 2.2. Chemical Reaction Synthesis of HMS Nanowire Arrays

**Figure 1:**
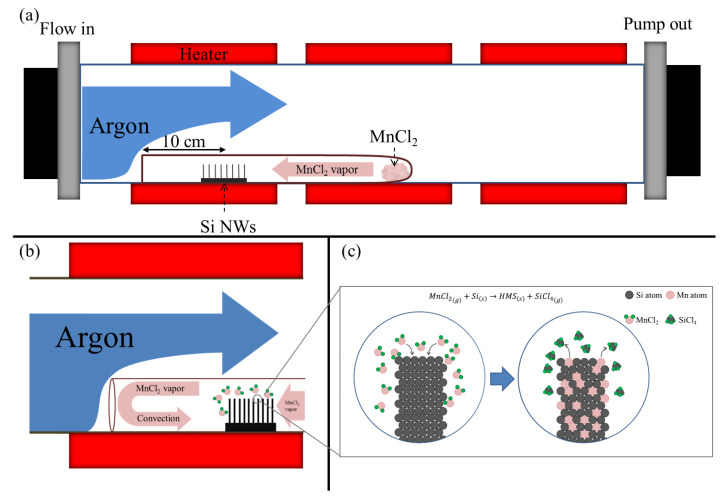
(**a**) Schematic illustration of the double tube system. (**b**) Argon flow and source vapor at the open end of the small tube. (**c**) Schematic illustration of the nanowire silicidation mechanism.

As shown in Figure 1a, the double tube system was composed of the home-made small test tube, which was 8 mm in diameter and 30 cm in length with one open end, being placed in the front section of the large tube, while the open end of the small tube faced toward the upstream flow direction. The as-prepared Si nanowire arrays and MnCl_2_ precursor were placed in the small tube. Argon gas was introduced into the furnace at 100 sccm and the ambient pressure was fixed at 0.45 torr. The furnace was annealed at 600–700 °C.

### 2.3. Characterization and Physical Properties of HMS NW Arrays

The morphology of nanowires was observed by field emission scanning electron microscopy (SEM, Hitachi SU8000, Tokyo, Japan). The structural characterization and phase identification of nanowires were completed with X-ray diffraction (XRD, RigakuD-max, Wilmington, MA, USA), energy dispersive spectroscopy (EDS) and spherical aberration corrected transmission electron microscopy (TEM, JEOL-ARM200F, Peabody, MA, USA). The NW arrays were then placed in a vacuum chamber equipped with a Keithley power supply for field emission measurements. The magnetic properties of the nanowires were conducted with superconducting quantum interference device (SQUID, Quantum Design MPMS, San Diego, CA, USA).

## 3. Results and Discussion

### 3.1. Sealing Effect of the Double Tube System

In the double tube system, as shown in Figure 1b, Ar gas flowed into the large tube from left to right, while the MnCl_2_ precursor evaporated at the reaction temperature diffused in the opposite direction from the closed end of the small tube. The argon and MnCl_2_ vapor met at the open end, contributing to the fact that the argon flowed above the small tube and impeded the MnCl_2_ vapor from flowing out of the small tube. The convection of the MnCl_2_ vapor at the open end of the small tube efficiently enhanced the MnCl_2_ vapor pressure in the small tube. We propose that this phenomenon was a sealing effect, improving the efficiency of the reaction between the MnCl_2_ vapor and Si nanowires. In our experience, the enhanced flow rate or reduced ambient pressure could improve the sealing effect in the double system due to the increasing velocity of the flowing argon. Notably, the distance between the substrate position and the open end of the small tube was proportional to the MnCl_2_ vapor pressure at the substrate position.

### 3.2. Dependence on Reaction Time at 600 °C

**Figure 2:**
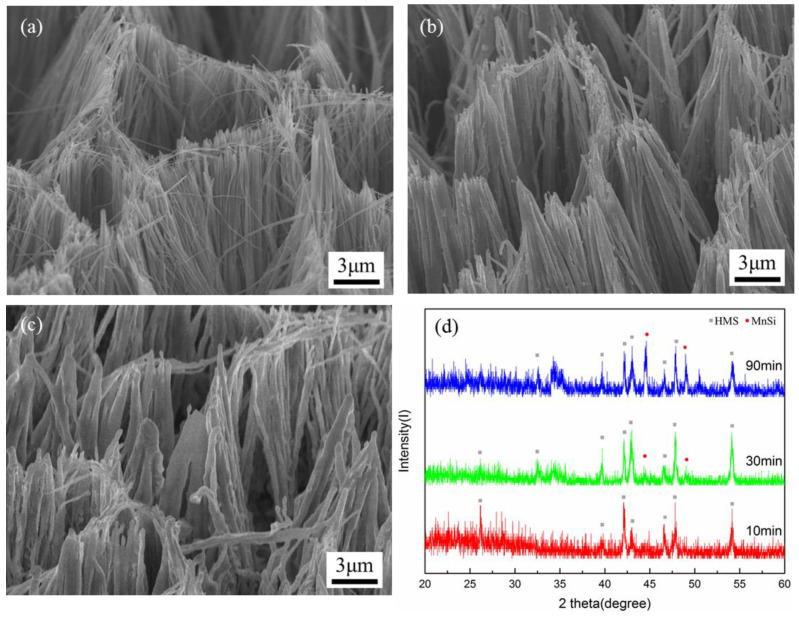
SEM images with a 45° tilt show the morphology of nanowire (NW) arrays with reaction duration of (**a**) 10 min, (**b**) 30 min and (**c**) 90 min at 600 °C, and (**d**) the corresponding XRD patterns obtained.

SEM images with a 45° tilt in Figure 2a–c show the Si nanowire arrays reacting with MnCl_2_ at 600 °C for 10, 30 and 90 min, respectively. XRD patterns of the samples for different reaction durations were obtained. The morphology and diameter were maintained during the 10 min of conversion from the as-prepared Si nanowires to the converted nanowires. When the reaction time was increased to 30 min, the nanowires were slightly widened in diameter and diffraction peaks of MnSi appeared. With the increasing reaction time, the nanowires became even thicker and the MnSi phase was observed clearly in the converted nanowires. It can be concluded that the Si nanowires which reacted with MnCl_2_ at the early stage of the reaction converted from Si to HMS phase. The nanowires underwent silicidation reaction through the pathway shown below:

(1) $${\mathrm{Si}}_{\left(s\right)}+{\mathrm{MnCl}}_{2}{}_{\left(g\right)}\to {\mathrm{HMS}}_{\left(s\right)}+{\mathrm{SiCl}}_{4}{}_{\left(g\right)}$$

As shown in Figure 1c, Mn atoms from the MnCl_2_ source reacted with the Si nanowires, leading to a byproduct of gaseous SiCl_4_. Afterwards, the SiCl_4_ reacted with the excess MnCl_2_ by the following equation:

(2) $${\mathrm{SiCl}}_{4}{}_{\left(g\right)}+{\mathrm{MnCl}}_{2}{}_{\left(g\right)}\to {\mathrm{MnSi}}_{\left(s\right)}+3{\mathrm{Cl}}_{2}{}_{\left(g\right)}$$

It was assumed that the reaction between the SiCl_4_ and MnCl_2_ contributed to the formation of MnSi around the surface of the HMS nanowires; thereby, more widening of the nanowires could be found. The results reveal that this could form a HMS-MnSi core-shell nanowire structure.

### 3.3. Dependence on Reaction Temperature

**Figure 3:**
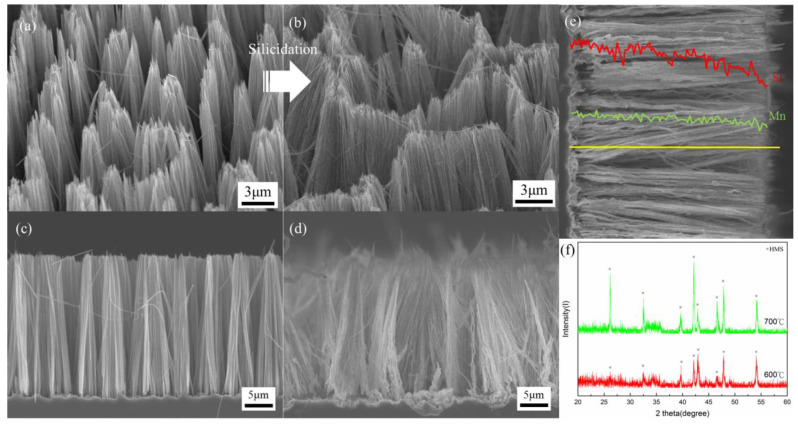
SEM images (**a**,**b**) with a 45°and 90° tilt show the morphology of (**a**,**c**) Si NW arrays and (**b**,**d**) converted higher manganese silicide (HMS) NW arrays at 700 °C for 20 min. (**e**) EDS concentration profile shows Si (denoted by red line) and Mn (denoted by green line) atomic percentage distribution along the axial direction of HMS NWs. (**f**) XRD patterns of the HMS nanowires at reaction temperatures of 600 °C and 700 °C were obtained, respectively.

In order to further study the reaction of the nanowire silicidation and achieve high quality HMS nanowires, we increased the reaction temperature of the silicidation process. After the reaction was completed at 700 °C for 20 min, the morphology of the nanowires standing on the substrate was observed by SEM with a 45° and 90° tilt in Figure 3a–d. The nanostructure of the converted nanowires was well maintained as compared with the initial Si nanowires. EDS studies show that the atomic ratio of Mn and Si along the axial direction of the converted nanowires was of high uniformity. XRD studies show that HMS appeared in the converted nanowires at 700 °C. Compared with the reaction temperature of 600 °C, the HMS major peaks of the converted nanowires at 700 °C were much stronger and the morphology of the nanowires was maintained. This indicates that higher reaction temperature facilitated silicidation and completed the reaction. Notably, MnCl_2_ vapor pressure was enhanced significantly at the reaction temperature of 700 °C, higher than the MnCl_2_ melting point of approximately 654 °C. In addition, no MnSi phase was found; it could not stably exist at 700 °C since the HMS phase was the stable phase at the higher temperature.

### 3.4. High Resolution Transmission Electron Microscopy

**Figure 4:**
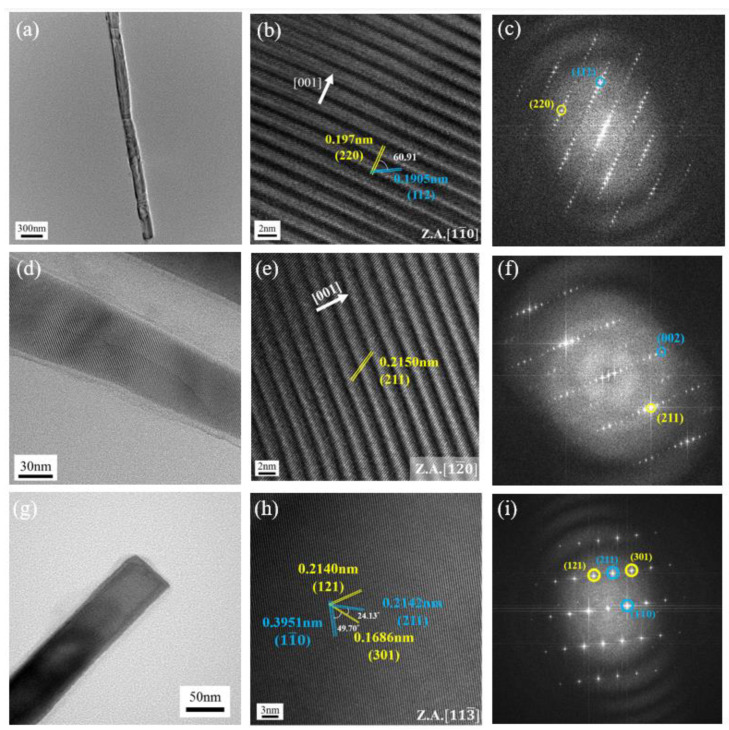
HRTEM images of HMS nanowires. (**a**,**d**,**g**) TEM images of HMS NWs. (**b**,**e**,**h**) Lattice-resolved HRTEM images with zone axis of (**b**) $\left(1\bar{1}0\right)$, (**e**) $\left(1\bar{2}0\right)$ and (**h**) $\left(11\bar{3}\right)$ and the corresponding FFT selected area diffraction patterns (**c**,**f**,**i**).

The lattice-resolved images of the converted nanowires were also studied using high resolution transmission electron microscopy (HRTEM). HRTEM images show that the nanowires were single crystalline silicide, and their corresponding fast Fourier transforms (FFTs) were indexed to NCL-structured HMS phase with some distinct features. The unusual lattice fringes in the HRTEM images of Figure 4b,e were from zone axis of $\left(1\bar{1}0\right)\;\mathrm{and}\;\left(1\bar{2}0\right)$, representing contrast modulation bands with long-range order along the c-direction of the crystal lattice. FFT images in Figure 4c,f showing closely spaced spots along the c-direction with spacing anomalies can be indexed to a tetragonal unit cell as labeled. These features emerge only when the zone axis is perpendicular to the crystallographic c-direction due to the mismatch of Mn and Si sublattices. As shown in Figure 4h, the HRTEM image with $\left(11\bar{3}\right)$ zone axis shows common lattice fringes measured to be 0.2140, 0.2142, 0.1686 and 0.3951 nm, corresponding to the interplanar spacings of (121), (211), (301) and $\left(1\bar{1}0\right)$ planes, confirming that the converted nanowire was HMS phase. There were no contrast modulation bands in the HRTEM with $\left(11\bar{3}\right)$ zone axis, since the zone axis was not perpendicular to the c-axis of the HMS lattice.

Each HMS NCL structure has a Si-sublattice which is slightly different in the c lattice parameter, contributing to a homologous series of HMS phases with close stoichiometry. Since the mismatch of the HMS phases of nanowires cannot be distinguished by XRD pattern and EDS spectrum easily, we followed a HMS phase identification method developed by Ye and Jin et al. [18], identifying two HMS nanowires fabricated at 700 °C for 20 min to be Mn_27_Si_47_ HMS phase. As previously reported, each distinct subphase of a homologous series of HMS phases has a unique physical property, and it was possible that the slight variation in Gibbs free energy or phase conversion in silicidation facilitated the formation of different subphases.

### 3.5. Field Emission Measurements of HMS Nanowires

**Figure 5:**
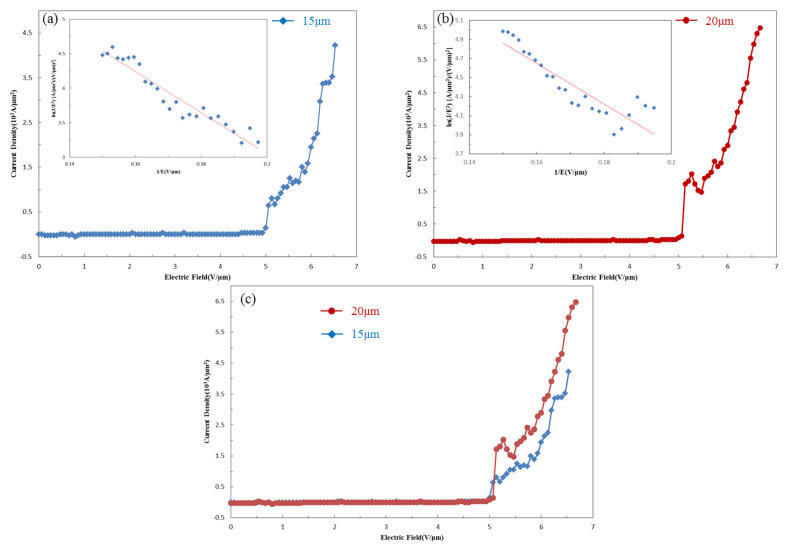
J-E plots for HMS nanowires with lengths of (**a**) 15 μm and (**b**) 20 μm. The insets are the corresponding ln (J/E^2^)-1/E plots. (**c**) Field enhancement comparison between nanowires of different lengths.

We investigated the field emission properties of the HMS nanowires fabricated at 700 °C in a vacuum chamber equipped with a Keithley power supply. The measured sample was sandwiched between two ITO (Indium tin oxide) layers at a pressure of approximately 10^−5^ torr. In addition, the distance between the anode and emitting top of the nanowire arrays substrate was 150 μm. Figure 5a,b show the field emission properties of HMS nanowires with different lengths of 15 and 20 um with insets of the corresponding ln(J⁄E^2^)-1/E plots. The field emission current density follows the equation derived from the Fowler–Nordheim relation:

(3) $$\ln\left(J/E^{2}\right)-\ln\left({A\beta }^{2}/\Phi \right)=-{B\Phi }^{3/2}/\beta E$$

where J is current density, Φ is material work function, E is applied electric field, β is the field enhancement factor and A and B are constants, A = 1.54 × 10^−10^ (AV^−2^ eV) and B = 6.83 × 10^3^ (eV^−3/2^ V μm^−1^). Based on the slope derived from the ln( J/E^2^)-1/E scatter plot, the field enhancement factors of the HMS nanowires with lengths of 15 and 20 μm were determined to be 2374 and 3307, respectively. The field enhancement factor was attributed to the metallic property, aspect ratio and morphology of the nanowire. The field emission results are great, having been rarely reported in the previous works related to HMS NWs.

### 3.6. Magnetic Properties of HMS Nanowires

**Figure 6:**
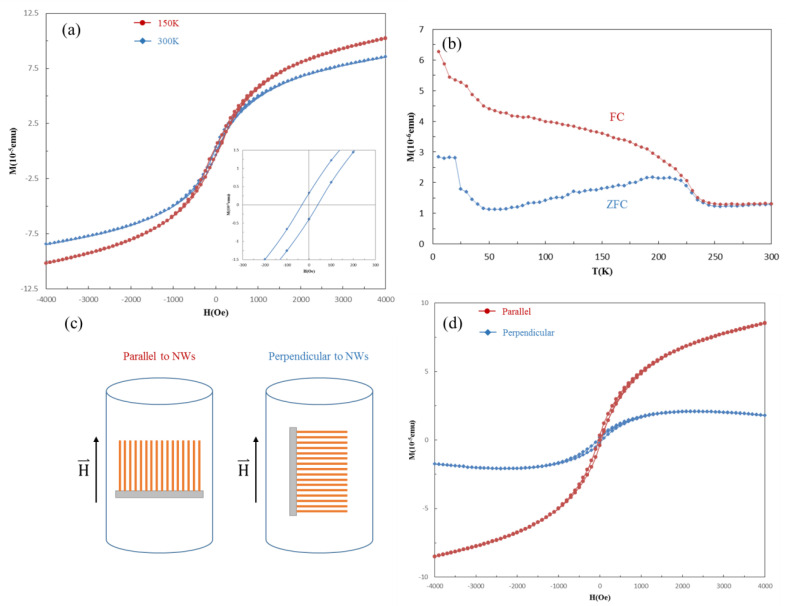
Magnetic properties of HMS nanowire arrays. (**a**) Hysteresis loops at 150 K and 300 K in M-H plots. (**b**) Temperature dependence of field cooled (FC) and zero-field cooled (ZFC) magnetization. (**c**) Schematic illustration of the relationship between magnetic field intensity and the axial direction of HMS NW arrays, and (**d**) hysteresis loops were obtained, respectively.

Since manganese silicides are excellent candidates to be applied in semiconductor nanodevices, we investigated the magnetic properties of HMS nanowires using a superconducting quantum interference device (SQUID, Quantum Design MPMS, San Diego, CA, USA) and vibrating sample magnetometer (VSM, Quantum Design MPMS, San Diego, CA, USA). Figure 6a shows that the hysteresis loops of HMS nanowire arrays can be found in the H-M plots by SQUID VSM at 150 K and 300 K. The saturation magnetization and magnetic susceptibility decreased with increasing temperature, while the hysteresis loops were still observed at room temperature. The magnetic feature revealed that the coercive field was about 50 Oe and the saturation magnetization was over 1 × 10^−4^ emu. Furthermore, the relationship between magnetism and temperature in the field cooled (FC) and zero-field cooled (ZFC) with a magnetic field of 500 Oe is shown in Figure 6b. The ZFC curve went up and then down with the increasing temperature, while the FC curve went down constantly with the increasing temperature. The difference between FC and ZFC curves shows that the HMS nanowire arrays were ferromagnetic and that the Curie temperature was over 300 K. Most magnetic properties of metal silicide nanostructures highly depend on the properties in bulk materials; however, there was a large enhancement of Curie temperature in the nanostructured HMS as compared with bulk. Additionally, we studied the relationship between the direction of the applied electric field and the axial direction of the standing nanowire arrays at 300 K. Figure 6c,d reveal the magnetic properties with different electric fields applied to the standing nanowire arrays. It can be found that with the electric field perpendicular to the standing nanowire arrays, there was lower saturation magnetization and magnetic susceptibility. Since the magnetic domains of the nanowires grew along the direction of the applied electric field, it was possible that the narrow space of the nanowires blocked the growth of the magnetic domains, leading to lower saturation magnetization and magnetic susceptibility.

## 4. Conclusions

In summary, a novel silicidation method was studied for the efficient synthesis of HMS nanowire arrays with a double tube CVD system using MnCl_2_ as a single source precursor. Single crystalline HMS nanowire arrays were fabricated at different reaction temperatures with a sealing effect and controlled Mn vapor pressure. The HMS nanowires were identified as Mn_27_Si_47_, a subphase of HMS, having Nowotny Chimney Ladder crystal structure. Field emission measurements indicate that the HMS nanowire arrays possessed outstanding field enhancement factors, making them promising candidates in future field emission display. Magnetic property measurements demonstrate the room temperature ferromagnetism of the HMS nanowire arrays with Curie temperature of over 300 K and high dependence on the relationship between the electric field direction and the axial direction of the nanowires.
